# La-Doped ZnO/SBA-15 for Rapid and Recyclable Photodegradation of Rhodamine B Under Visible Light

**DOI:** 10.3390/molecules30244800

**Published:** 2025-12-16

**Authors:** Ziyang Zhou, Weiye Yang, Jiuming Zhong, Hongyan Peng, Shihua Zhao

**Affiliations:** 1 College of Physics and Electronic Engineering, Hainan Normal University, Haikou 571158, China; 202312070200014@hainnu.edu.cn (Z.Z.); haha_weiye@163.com (W.Y.); mdjphy@163.com (H.P.); 2 The Innovation Platform for Academicians of Hainan Province, Haikou 571158, China

**Keywords:** zinc oxide, SBA-15, photocatalysis, Rhodamine B

## Abstract

La-doped ZnO nanoclusters confined within mesoporous SBA-15 were synthesized using an impregnation–calcination method and evaluated for their visible-light-driven photocatalytic degradation of Rhodamine B (RhB). Small-angle X-ray diffraction (XRD) and transmission electron microscopy (TEM) confirmed the preservation of the 2D hexagonal mesostructure of SBA-15 post-loading. In contrast, wide-angle XRD and Fourier-transform infrared spectroscopy (FT-IR) analyses revealed that the incorporated ZnO existed predominantly as highly dispersed amorphous or ultrafine clusters within the mesopores. N_2_ adsorption–desorption measurements exhibited Type IV isotherms with H1 hysteresis loops. Compared to pristine SBA-15, the specific surface area and pore volume of the composites decreased from 729.35 m^2^ g^−1^ to 521.32 m^2^ g^−1^ and from 1.09 cm^3^ g^−1^ to 0.85 cm^3^ g^−1^, respectively, accompanied by an apparent increase in the average pore diameter from 5.99 nm to 6.55 nm, attributed to non-uniform pore occupation. Under visible-light irradiation, the photocatalytic performance was highly dependent on the La doping level. Notably, the 5% La-ZnO/SBA-15 sample exhibited superior activity, achieving over 99% RhB removal within 40 min and demonstrating the highest apparent rate constant (k = 0.1152 min^−1^), surpassing both undoped ZnO/SBA-15 (k = 0.0467 min^−1^) and other doping levels. Reusability tests over four consecutive cycles showed a consistent degradation efficiency exceeding 93%, with only a ~7 percentage-point decline, indicating excellent structural stability and recyclability. Radical scavenging experiments identified h^+^, ·OH, and ·O_2_^−^ as the primary reactive species. Furthermore, photoluminescence (PL) quenching observed at the optimal 5% La doping level suggested suppressed radiative recombination and enhanced charge carrier separation. Collectively, these results underscore the synergistic effect of La doping and mesoporous confinement in achieving fast, efficient, and recyclable photocatalytic degradation of organic pollutants.

## 1. Introduction

With the acceleration of global industrialization, the effective treatment of organic pollutants, such as dye-contaminated wastewater, has become a significant challenge in environmental science. Within the broad spectrum of wastewater remediation approaches, photocatalytic oxidation is recognized as one of the most promising approaches for water pollution control due to its capacity to utilize solar energy for the thorough mineralization of pollutants, alongside its inherent environmental compatibility and sustainability [1,2]. Among semiconductor photocatalysts, ZnO has attracted considerable attention due to its superior photocatalytic performance, excellent chemical stability, and low cost [3,4]. However, its practical application is hindered by two primary limitations: (i) the rapid recombination of photogenerated e^−^–h^+^ pairs, which substantially diminishes quantum efficiency [5], and (ii) the aggregation of nanoscale particles, which significantly impairs the accessibility of active sites and mass transfer kinetics [6].

To address these limitations, a synergistic modification approach that integrates carrier confinement with elemental doping has been developed [7,8,9]. Ordered mesoporous silica SBA-15 is an exemplary support for confining metal oxide nanoparticles due to its highly regular 2D hexagonal pore structure (5–10 nm), exceptionally large surface area (>600 m^2^ g^−1^), and significant thermal stability [10,11,12]. This distinctive pore confinement effect not only effectively inhibits the aggregation and leaching of active components but also preserves efficient mass transfer pathways. This strategy has been demonstrated to be effective in various catalytic systems, including TiO_2_ and Fe_2_O_3_ [13,14,15].

Concurrently, doping with rare-earth elements constitutes an effective strategy for modifying the electronic structures of semiconductors. This approach enhances optoelectronic properties by introducing defect energy levels and altering local charge distributions [16]. La, a representative rare-earth element, has been demonstrated to extend the visible-light absorption range of ZnO and inhibit carrier recombination through the formation of electron trapping centers, thereby improving overall photocatalytic efficiency [8,17].

Building upon this foundation, the research synthesized La-ZnO/SBA-15 composite photocatalysts using an impregnation–calcination method to improve light absorption and charge separation by confining La-doped ZnO nanoclusters within the mesopores of SBA-15. Comprehensive characterization techniques, including X-ray diffraction (XRD), transmission electron microscopy (TEM), Fourier-transform infrared spectroscopy (FT-IR), and N_2_ adsorption–desorption analysis, were employed alongside Rhodamine B (RhB) degradation experiments, kinetic analyses, and radical trapping studies. These investigations demonstrated that the La doping ratio significantly impacts photocatalytic activity. Importantly, the confined active species (e^−^, h^+^, ·OH, and ·O_2_^−^) engage in synergistic oxidation pathways during RhB degradation. This study provides a viable design framework for high-performance, recyclable mesopore-confined photocatalysts with significant potential applications in environmental remediation.

## 2. Results

### 2.1. Analysis of Structure and Composition via XRD

**Figure 1:**
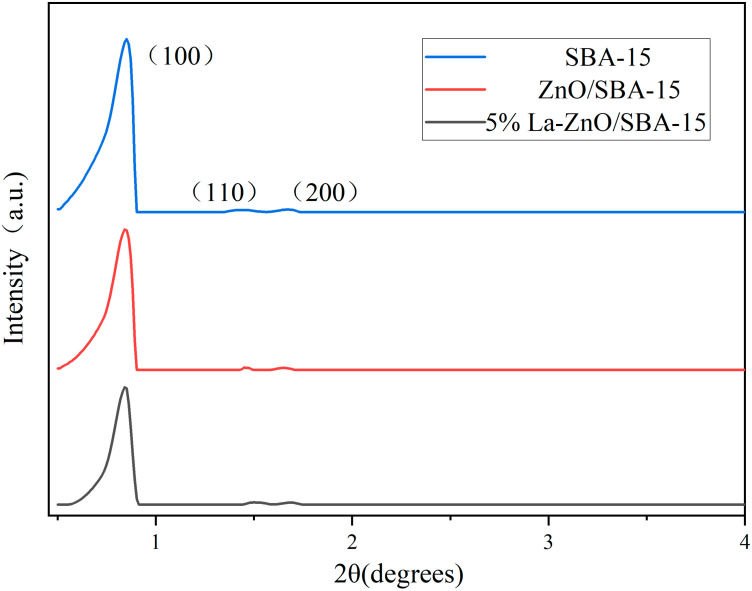
SAXRD patterns of the prepared samples.

Figure 1 illustrates the small-angle X-ray diffraction (SAXRD) patterns of the synthesized SBA-15 support, ZnO/SBA-15, and 5% La-doped ZnO/SBA-15 composites. The SBA-15 material exhibits three diffraction peaks at 2θ values of 0.84°, 1.45°, and 1.68°, which correspond to the (100), (110), and (200) reflections characteristic of highly ordered hexagonal mesoporous silica SBA-15 [10]. The SAXRD patterns of both ZnO/SBA-15 and 5% La-doped ZnO/SBA-15 exhibit identical characteristic peaks, confirming that the hexagonal mesostructure remains intact following impregnation and calcination. This observation demonstrates that the incorporation of ZnO and subsequent La doping does not disrupt the 2D hexagonal mesoscopic order, thereby highlighting the excellent thermal stability of SBA-15 as a catalyst support.

**Figure 2:**
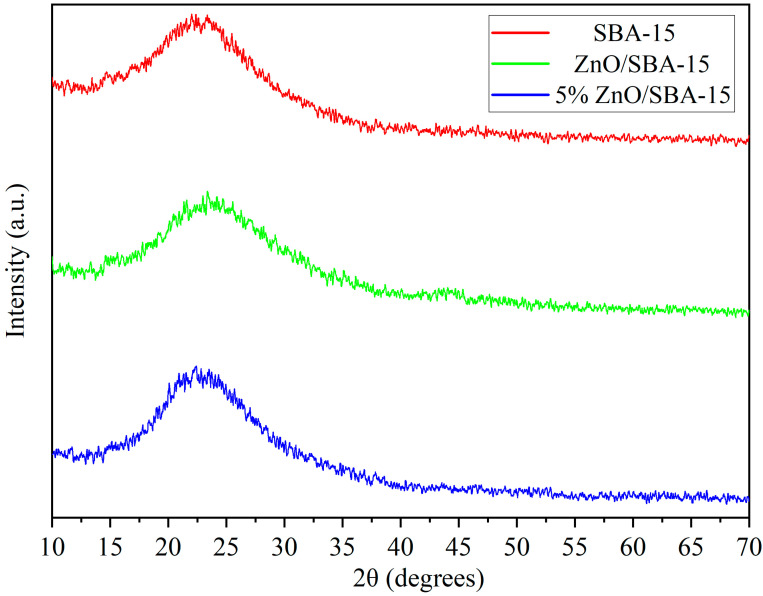
WAXRD patterns of the prepared samples.

Figure 2 depicts the wide-angle XRD (WAXRD) patterns of the SBA-15 support, ZnO/SBA-15, and 5% La-ZnO/SBA-15 composites. All samples exhibit a broad diffraction hump in the 16–33° (2θ) range, characteristic of the amorphous silica framework of SBA-15 [18,19,20,21]. No additional reflections attributable to crystalline ZnO are observed for the composites, even though the Zn(NO_3_)_2_ precursor is completely decomposed to ZnO during calcination at 550 °C [22]. This absence of distinct ZnO peaks indicates that the ZnO phase is present as highly dispersed, ultrafine nanoclusters confined within the SBA-15 mesopores, with crystallite sizes below the XRD detection limit (~3–5 nm) and with weak reflections overlapped by the intense amorphous silica [23,24]. Such pore-confined ZnO nanoclusters are often described as “XRD-amorphous” in SBA-15-based systems [25,26].

### 2.2. HRTEM Analysis

**Figure 3:**
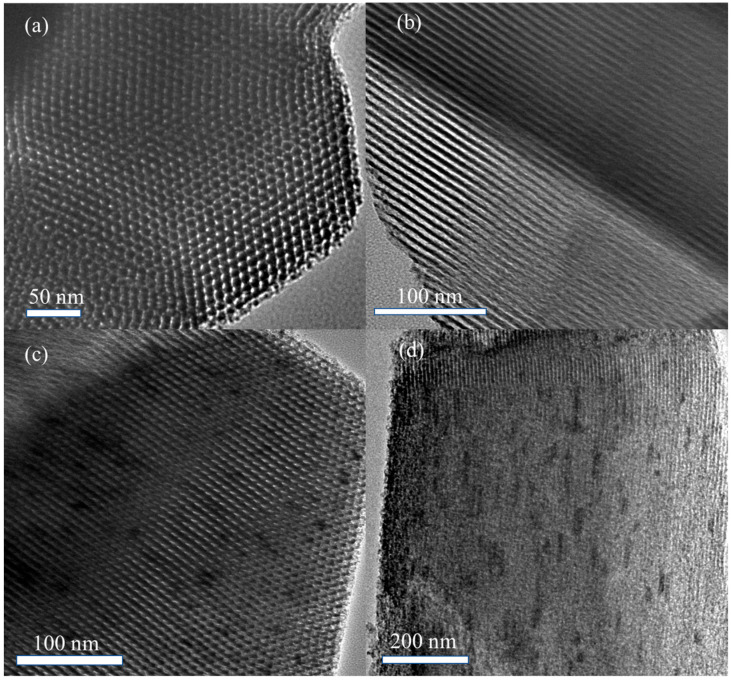
HRTEM images of SBA-15 (**a**,**b**) and 5% La-ZnO/SBA-15 (**c**,**d**): (**a**) SBA-15 perpendicular to pore channels; (**b**) SBA-15 parallel to pore channels; (**c**) 5% La-ZnO/SBA-15 perpendicular to pore channels; (**d**) 5% La-ZnO/SBA-15 parallel to pore channels.

Figure 3 presents high-resolution transmission electron microscopy (HRTEM) images of SBA-15 and 5% La-ZnO/SBA-15. Figure 3a illustrates the TEM image of SBA-15 viewed perpendicular to the pore channels, revealing a highly ordered 2D hexagonal honeycomb pore arrangement. The channels exhibit a regular configuration with long-range periodicity, uniform pore size distribution (~5–7 nm), and structural characteristics typical of SBA-15 mesostructures [27]. Figure 3b depicts the image taken parallel to the pore axis, illustrating straight, parallel channels with consistent spacing and high aspect ratios, thereby further confirming the structural regularity and integrity.

Following the loading of the active component, the 5% La-ZnO/SBA-15 composite preserves the mesoporous structure of SBA-15, in agreement with the SAXRD analysis. In Figure 3c, the composite exhibits a well-defined honeycomb pore arrangement characterized by hexagonal ordering and different channel boundaries. Within certain pores, discrete dark spots are observed, which are likely attributable to the incorporated ZnO species. The image oriented parallel to the pore direction (Figure 3d) shows numerous dispersed dark nanoparticles on the pore walls and within the channels, presumably corresponding to ZnO clusters formed through the impregnation-annealing process.

### 2.3. FT-IR Analysis

**Figure 4:**
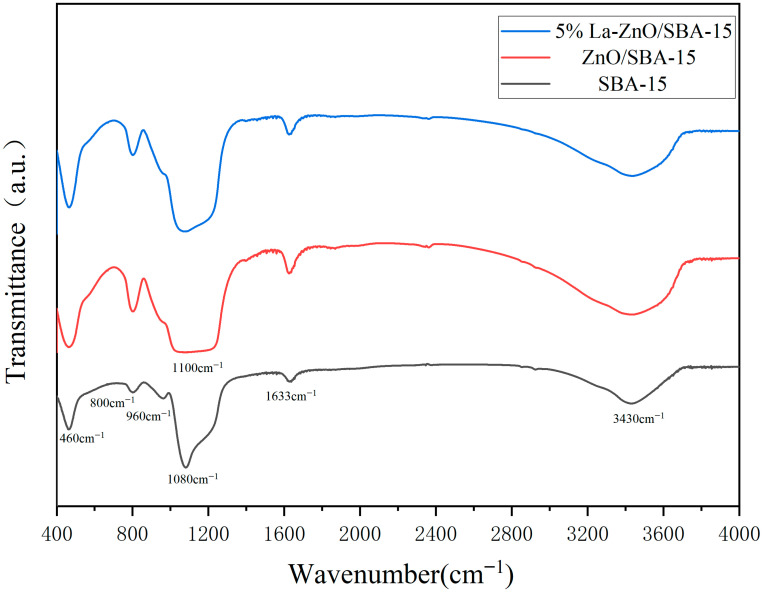
FT-IR spectra of the SBA-15, ZnO/SBA-15, and 5% La-ZnO/SBA-15.

Figure 4 illustrates the FT-IR spectra of SBA-15, ZnO/SBA-15, and 5% La-ZnO/SBA-15. All samples exhibit absorption peaks at approximately 3430 cm^−1^ and 1633 cm^−1^, which correspond to the stretching vibration of surface-adsorbed water or silanol groups (Si–OH) and the bending vibration of H–O–H in water molecules, respectively [28,29]. Within the skeletal vibration region (1400–400 cm^−1^), the broad absorption band near 1080 cm^−1^ is attributed to the asymmetric stretching vibration of Si–O–Si [30], whereas the peaks at 800 cm^−1^ and 460 cm^−1^ are assigned to the symmetric stretching and bending vibrations of Si–O–Si, respectively [18,31]. Additionally, a weak absorption band near 960 cm^−1^ is observed in all samples, which is typically ascribed to the stretching vibration of Si–O–Si [32].

In the ZnO/SBA-15 and 5% La-ZnO/SBA-15 samples, a broad absorption band ranging from 1020 cm^−1^ to 1230 cm^−1^ is observed, with the signal near 1110 cm^−1^ attributed to the stretching vibration of Zn–O bonds, which is typically characteristic of amorphous or polycrystalline ZnO materials [33]. Notably, no additional different characteristic absorption peaks of ZnO are detected beyond this broad band, suggesting that ZnO is highly dispersed within the SBA-15 support, and likely to exist in an amorphous form or as ultrafine clusters. The weak infrared signal may result from the low ZnO content, which is potentially obscured by the intense Si–O–Si skeletal vibrations of the SBA-15 matrix, or from the high dispersion of ZnO clusters that prevents the formation of well-defined absorption features.

### 2.4. Sorption Analysis

**Figure 5:**
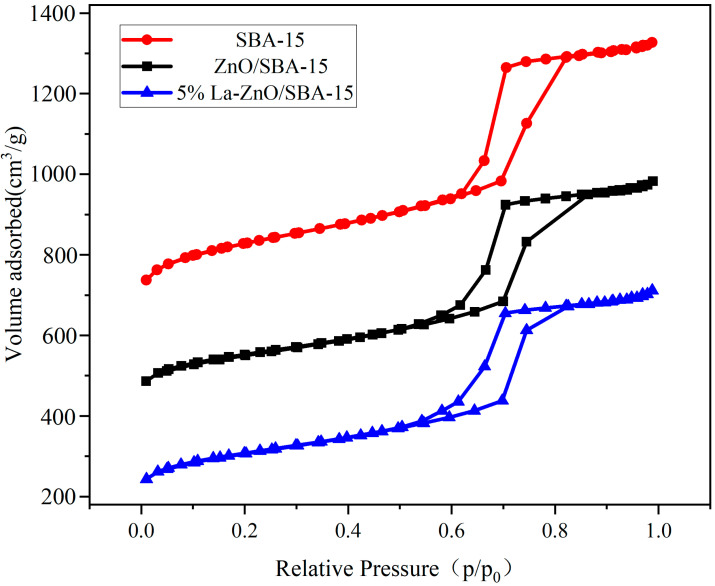
N_2_ adsorption–desorption isotherms of the SBA-15, ZnO/SBA-15, and 5% La-ZnO/SBA-15.

Figure 5 depicts the N_2_ adsorption–desorption isotherms for SBA-15, ZnO/SBA-15, and 5% La-ZnO/SBA-15. All samples exhibit Type IV isotherms according to the 2015 IUPAC classification [34], accompanied by H1-type hysteresis loops, which are indicative of materials possessing narrow pore size distributions and uniform cylindrical mesopores [35]. The absence of significant changes in the isotherm type suggests that the ordered mesoporous channels of SBA-15 remain largely preserved during the formation of ZnO clusters, confirming the results obtained from SAXRD and TEM analyses.

In Table 1, the structural parameters exhibit significant changes following ZnO loading. Specifically, the BET surface area (S_BET_) decreases from 729.35 m^2^ g^−1^ for pure SBA-15 to 521.32 m^2^ g^−1^, representing a reduction of approximately 29.5%. Similarly, the total pore volume (Vp) decreases from 1.09 cm^3^ g^−1^ to 0.85 cm^3^ g^−1^, corresponding to a reduction of approximately 22.0%. These results confirm the successful incorporation of ZnO into the SBA-15 framework, with partial occupation of the pore channels resulting in moderate pore blockage.

**Figure 6:**
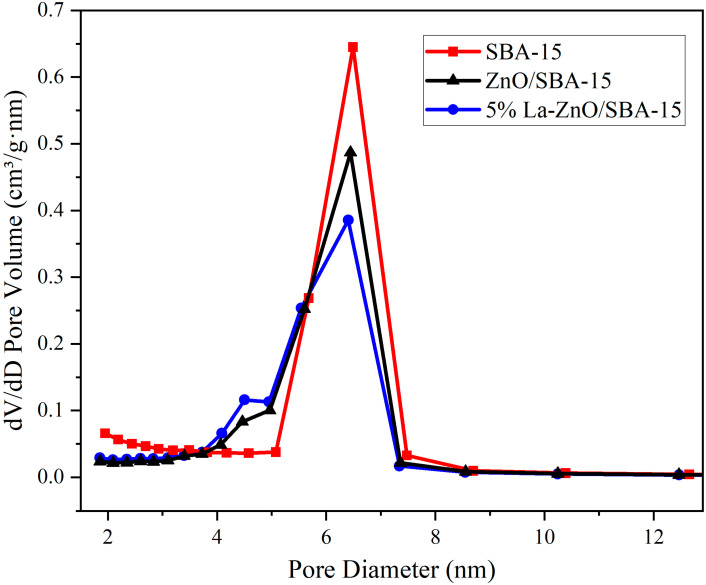
BJH pore size distribution curves of the SBA-15, ZnO/SBA-15, and 5% La-ZnO/SBA-15.

Despite a reduction in pore volume, the average pore diameter calculated using the BJH method increases from 5.99 nm to 6.55 nm. This observation is further clarified by the BJH pore size distribution curves in Figure 6, which show that the curves following ZnO loading exhibit broadened peaks and decreased intensity. These changes indicate a broader pore size distribution and reduced structural homogeneity. This phenomenon results from the non-uniform deposition of ZnO precursors within the SBA-15 channels: during impregnation and calcination, ZnO nanoparticles preferentially nucleate at constricted sites or pore entrances, thereby blocking smaller channels while leaving larger pores relatively accessible. Consequently, N_2_ desorption measurements predominantly reflect the larger pores, leading to an “apparent increase” in the average pore diameter. Furthermore, secondary high-temperature calcination may cause localized sintering of the SBA-15 silica framework or minor collapse of pore walls, further contributing to structural disorder.

Integrated analysis of TEM, XRD, FT-IR, and N_2_ adsorption–desorption data reveals that the synthesized ZnO/SBA-15 nanocomposite retains the ordered hexagonal mesostructure of SBA-15 while confining ZnO clusters in an amorphous state within the mesopores. This pore-confined loading strategy not only suppresses ZnO nanoparticle aggregation—enhancing exposure of active sites—but also preserves high surface area and interconnected pore channels, facilitating reactant diffusion and mass transfer during catalytic processes. Such structural features provide a robust foundation for efficient photocatalytic degradation of organic pollutants in water, aligning with established literature on mesoporous silica-encapsulated metal oxides for environmental catalysis.

## 3. Discussion

### 3.1. Comparison of Photocatalytic Activity

**Figure 7:**
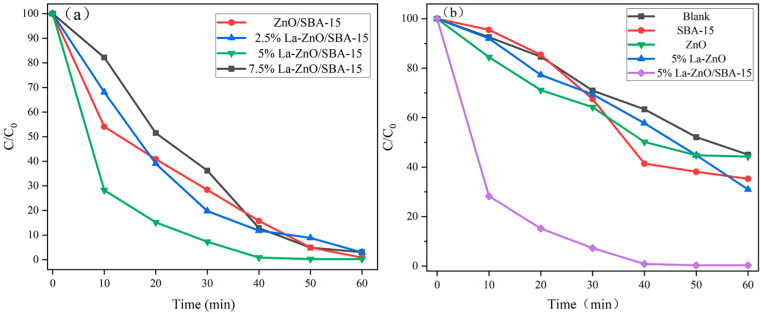
(**a**) Photocatalytic degradation of RhB over ZnO/SBA-15 samples with different La doping ratios under visible-light irradiation. (**b**) Photocatalytic degradation of RhB over blank solution, pure SBA-15, pure ZnO, La-ZnO, and 5% La-ZnO/SBA-15 under visible-light irradiation.

Figure 7a illustrates the photocatalytic degradation performance of RhB under visible light irradiation for ZnO/SBA-15 samples with varying La doping ratios. The undoped ZnO/SBA-15 sample achieves approximately 98% RhB degradation within 60 min, indicating favorable intrinsic photocatalytic activity. However, increasing the La doping concentration does not result in a linear improvement in photocatalytic performance: both the 2.5% and 7.5% La-doped ZnO/SBA-15 samples exhibit degradation efficiencies of approximately 95% at 60 min, comparable to the undoped sample. In contrast, the 5% La-doped ZnO/SBA-15 sample demonstrates significantly enhanced activity, achieving near-complete RhB degradation (>99%) within 40 min, thereby significantly outperforming all other samples.

The blank solution, pure SBA-15, pure ZnO, and La-ZnO exhibit nearly identical degradation efficiencies, achieving only ~40% RhB degradation after 60 min of irradiation. In contrast, the 5% La-ZnO/SBA-15 composite achieves near-complete RhB degradation (>99%) within 40 min, exhibiting significantly superior performance compared to all other samples.

**Figure 8:**
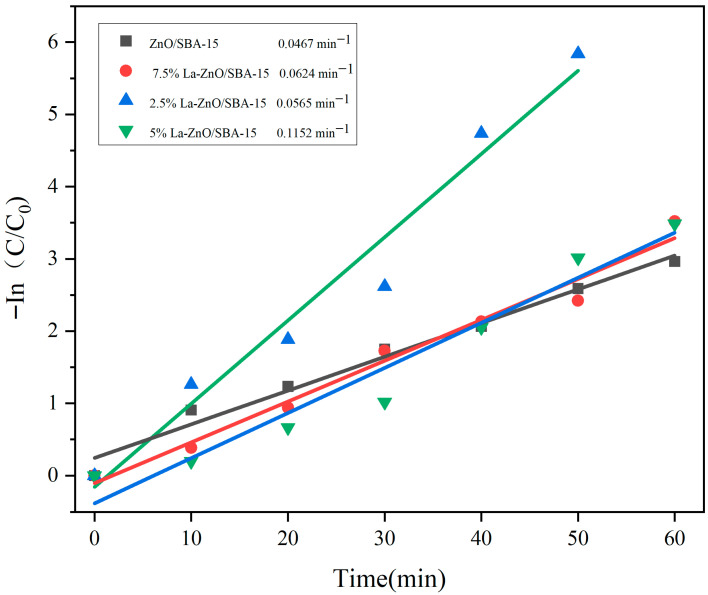
Pseudo-first-order kinetic fitting curves for RhB photodegradation over ZnO/SBA-15 samples with different La doping concentrations.

Figure 8 depicts the kinetic analysis based on the Langmuir–Hinshelwood model, demonstrating that the photodegradation of RhB adheres to pseudo-first-order kinetics [36]:

In(C_0_/C) = −kt (1)

where C_0_ and C represent the initial and instantaneous concentrations of RhB at time t, respectively; k represents the apparent first-order rate constant (min^−1^); and t represents the reaction time (min).

The fitting results indicate rate constants of 0.1152 min^−1^ for 5% La-ZnO/SBA-15, 0.0467 min^−1^ for undoped ZnO/SBA-15, 0.0565 min^−1^ for 2.5% La-ZnO/SBA-15, and 0.0624 min^−1^ for 7.5% La-ZnO/SBA-15. These results reveal that 5% La-ZnO/SBA-15 exhibits a significantly higher rate constant, thereby confirming its superior kinetic performance in the photodegradation of RhB.

**Figure 9:**
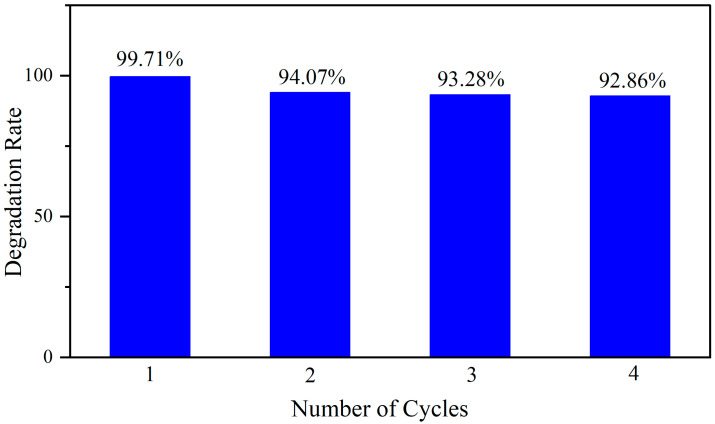
Cycling degradation of RhB by the 5% La-ZnO/SBA-15 under visible light irradiation.

Figure 9 presents the recyclability test results of 5% La-ZnO/SBA-15 over four consecutive cycles. In the initial run, the catalyst achieved 99% degradation of RhB, indicating excellent initial activity. During the second cycle, the efficiency remained at 94%, demonstrating robust structural stability. The degradation efficiency stabilized at approximately 93% in the third and fourth cycles, with no significant decline, thereby confirming consistent catalytic performance. Although minor deactivation of active sites or slight leaching of active components may have occurred during recycling, the overall performance exhibited no substantial deterioration. After four cycles, efficiency decreased by approximately 7%, underscoring the catalyst’s excellent reusability and operational stability for practical applications. As summarized in Table 2, the photocatalytic performance of the synthesized 5% La-ZnO/SBA-15 composite is compared with various ZnO-based catalysts reported in the literature for dye degradation. The comparison reveals that the 5% La-ZnO/SBA-15 composite achieves a high degradation efficiency (>99%) within a notably shorter time (40 min) under visible light. For instance, the ZnO–SiO_2_ composite [37] required 60 min to reach a comparable efficiency under UV light, and the flower-like ZnO@SiO_2_ [38] attained only 85% degradation after 180 min of UV light. These results indicate advantage and potential of the 5% La-ZnO/SBA-15 composite for efficient pollutant degradation under visible light.

### 3.2. Photocatalytic Mechanism Explained

**Figure 10:**
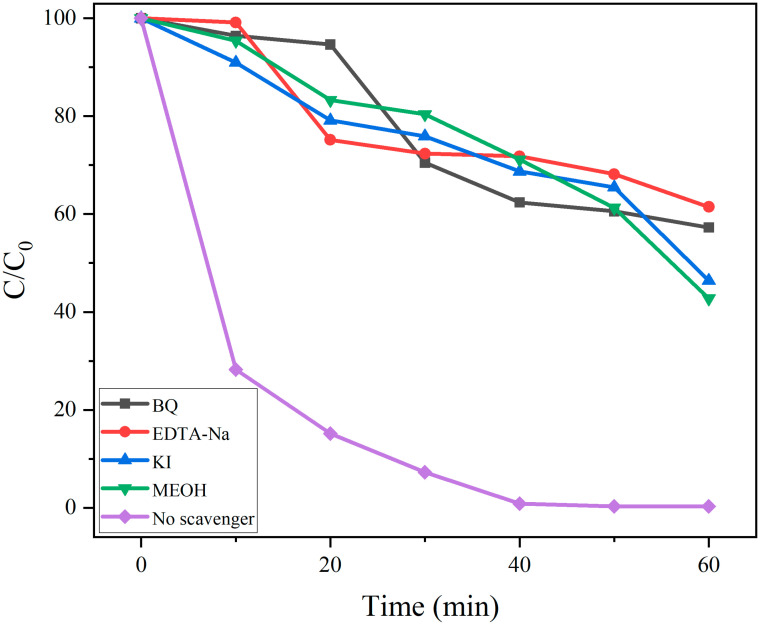
The degradation curve of RhB by 5% La-ZnO/SBA-15 photocatalyst under different quenching conditions.

To elucidate the reaction mechanism underlying the photodegradation of RhB over 5% La-ZnO/SBA-15, 50 mg of photocatalyst was dispersed in 100 mL of RhB solution (20 mg L^−1^), and systematic radical trapping experiments were performed (Figure 10). The addition of specific scavengers significantly inhibited the degradation efficiency: the ·O_2_^−^ scavenger BQ decreased the degradation rate to 32%; ·OH and h^+^ co-scavenger MeOH reduced it to 38%; the h^+^-specific scavenger EDTA-Na lowered it to 42%; and the e^−^ scavenger KI diminished it to 53%. In contrast, the degradation efficiency exceeded 99% in the absence of scavengers. These results unequivocally demonstrate the synergistic involvement of e^−^, h^+^, ·OH, and ·O_2_^−^ in the photodegradation process.

**Figure 11:**
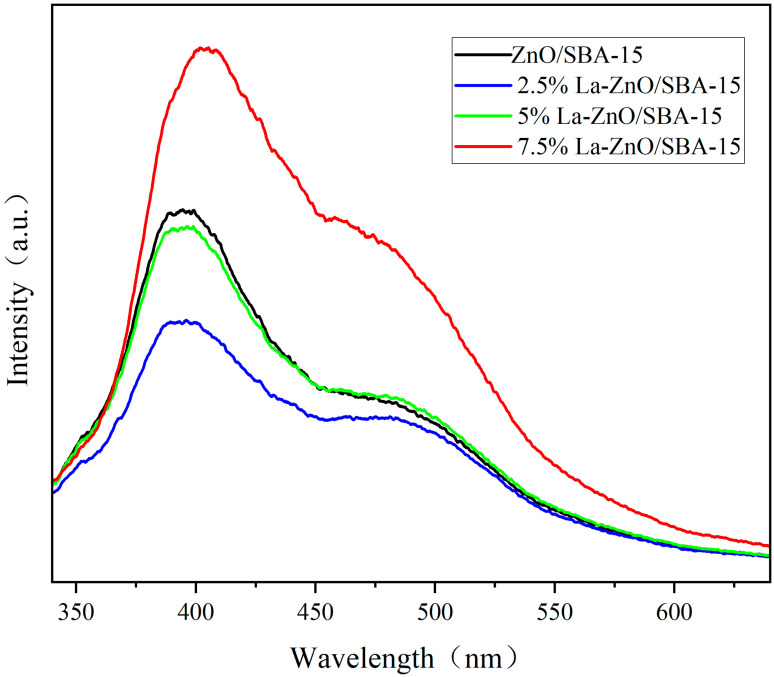
PL spectra of La-ZnO/SBA-15 composites.

To elucidate the charge carrier separation behavior underlying the radical trapping experiments, Photoluminescence (PL) spectroscopy was utilized to assess charge separation efficiency. In Figure 11, ZnO/SBA-15 samples with varying La doping concentrations exhibit two characteristic emission bands: a prominent broad peak near 380 nm and a weaker broad peak approximately 480 nm, corresponding to near-band-edge (NBE) radiative recombination and deep-level emission (DLE) associated with oxygen vacancies and Zn interstitial defects, respectively [43,44,45]. With increasing La doping concentration, the overall PL intensity first decreases and then increases: the undoped ZnO/SBA-15 sample shows the highest emission intensity, which decreases progressively at 2.5% and 5% La, and rises again at 7.5% La. The most pronounced fluorescence quenching is observed for the 5% La–ZnO/SBA-15 sample, indicating that this doping level provides an optimal concentration of La-induced defect states and trapping sites to promote the separation and migration of photogenerated e^−^–h^+^ pairs while suppressing their radiative recombination [46,47,48]. At higher La loading (7.5%), excessive La-related defects and/or La-rich clusters are likely formed, which act as additional recombination centers and partly offset the beneficial effect of La doping, in agreement with the partial recovery of PL intensity. The optimized charge-carrier separation at 5% La doping allows a larger fraction of photogenerated carriers to participate in surface redox reactions, thereby enhancing the photocatalytic degradation of RhB [49,50].

## 4. Materials and Methods

### 4.1. Materials

The following chemicals were utilized in the synthesis of the photocatalyst: La(NO_3_)_3_·6H_2_O (99%, Macklin Biochemical, Shanghai, China) and Zn(NO_3_)_2_·6H_2_O (99%, Macklin Biochemical, Shanghai, China) served as the La and Zn precursors, respectively; deionized water (DI), EO_20_PO_70_EO_20_ (P123, Mn ~5800, Sigma-Aldrich, St. Louis, MO, USA), tetraethyl orthosilicate (TEOS, 99%, Sigma-Aldrich, St. Louis, MO, USA), and HCl (37%, Sinopharm, Shanghai, China) were employed for the preparation of the SBA-15 template; and CH_3_CH_2_OH (99%, Sinopharm, Shanghai, China) was also used. All chemicals were utilized as received without further purification.

### 4.2. Synthesis of SBA-15

SBA-15 mesoporous material was synthesized with minor modifications to a previously established protocol [10]. In brief, 4 g of Pluronic P123 copolymer was added directly to 150 mL of 1.7 mol L^−1^ HCl solution and stirred in a water bath at 303 K until a colorless, transparent solution was obtained. The temperature of the bath then increased to 312 K, and 9.4 mL of TEOS was slowly added dropwise using a pipette. The mixture was continuously stirred at this temperature for 24 h. Subsequently, the solution was transferred to an autoclave and subjected to hydrothermal crystallization at 373 K for 24 h. Following the reaction, the product was collected via vacuum filtration using a Büchner funnel, and the resulting filter cake was alternately washed with deionized water and CH_3_CH_2_OH for three cycles to remove residual HCl and Pluronic P123 template. The solid was subsequently dried at 373 K for 12 h in air, followed by calcination at 823 K for 3 h in air, yielding SBA-15.

### 4.3. Synthesis of La-ZnO/SBA-15

**Figure 12:**
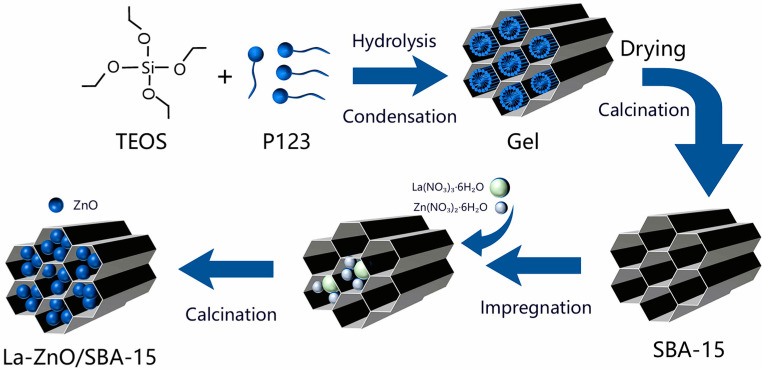
Schematic illustration of the preparation route of La–ZnO/SBA-15.

La-ZnO/SBA-15 composites were synthesized using a post-impregnation technique. SBA-15 was first vacuum-dried at 343 K for 2 h. Subsequently, a predetermined amount of La(NO_3_)_3_·6H_2_O and 0.24 g of Zn(NO_3_)_2_·6H_2_O were dissolved in 10 mL of CH_3_CH_2_OH, followed by the addition of 0.2 g of the pre-treated SBA-15. The resulting mixture was uniformly dispersed with ultrasonic assistance and continuously stirred at ambient temperature for 24 h. After stirring, the mixture was dried in air at 333 K for 12 h to ensure complete solvent evaporation, followed by annealing in a tube furnace at 823 K for 2 h. Employing this procedure, a series of La–ZnO/SBA-15 samples with different La-doping levels (0, 2.5, 5.0, and 7.5 at.%) were prepared by adjusting the amount of La(NO_3_)_3_ precursor relative to Zn(NO_3_)_2_ in the impregnation solution. Here, the La content is expressed as atomic percent (at.%), defined as the molar fraction of La in the total amount of La and Zn. The synthesis route is schematically illustrated in Figure 12.

### 4.4. Characterization

The surface morphology of the samples was examined using a FEI Tecnai G2 F20 TEM (FEI Company, Hillsboro, OR, USA). The crystal structure and mesostructure were investigated employing a Bruker D8 Advance XRD with Cu Kα radiation (λ = 1.5406 Å). FT-IR spectra were obtained using a Nicolet 6700 spectrometer (Thermo Fisher Scientific, Waltham, MA, USA) using the KBr pellet method, with a resolution of 4 cm^−1^. N_2_ adsorption–desorption isotherms were measured using a Micromeritics ASAP 2460 surface area analyzer (Micromeritics Instrument Corporation, Norcross, GA, USA). Prior to analysis, samples were degassed under vacuum at 373 K for 12 h. The specific surface area was calculated by the Brunauer–Emmett–Teller (BET) method. The pore-size distribution was obtained from the desorption branch of the isotherms by applying the Barrett–Joyner–Halenda (BJH) method, which uses the Kelvin equation to relate the relative pressure of capillary condensation to the mesopore radius. PL spectra were recorded with the fluorescence module of a HORIBA micro-Raman spectrometer, employing an excitation wavelength of 325 nm.

### 4.5. Photocatalytic Evaluation

The photocatalytic degradation of RhB at a concentration of 20 mg L^−1^ was investigated using La-ZnO/SBA-15 catalysts under irradiation from a 250 W Hg lamp. In the experimental procedure, 50 mg of the photocatalyst was dispersed in 100 mL of the RhB solution, followed by a 30 min dark adsorption period to achieve adsorption equilibrium. Subsequently, at 15 min intervals after the initiation of illumination, 3 mL aliquots were withdrawn and centrifuged to remove catalyst particles. The concentration of RhB was quantified by measuring the absorbance at 553 nm using a Hitachi U-3900 UV-Vis spectrophotometer.

The degradation efficiency was determined as follows:

Degradation (%) = (C_0_ − C_t_)/C_0_ × 100 (2)

To elucidate the active species involved in the photocatalytic process, radical scavenging experiments were conducted using specific quenchers: benzoquinone (BQ, 0.01 mol L^−1^) to target ·O_2_^−^, ethylenediaminetetraacetic acid disodium salt (EDTA-Na, 0.1 mol L^−1^) for h^+^, KI (0.1 mol L^−1^) for surface-bound ·OH and h^+^, and methanol (MeOH, 0.1 mol L^−1^) for ·OH [18,19,20,21].

## 5. Conclusions

La-doped ZnO/SBA-15 mesoporous composites were prepared by an impregnation–calcination route and evaluated as visible-light photocatalysts for RhB degradation. Structural characterization confirms that the ordered 2D hexagonal mesostructure and mesoporosity of SBA-15 are preserved after ZnO and La loading, with ZnO present as amorphous or ultrafine clusters confined within the mesopores.

Photocatalytic tests show that the activity strongly depends on the La content, with the 5% La-ZnO/SBA-15 sample exhibiting the best performance: it achieves nearly complete RhB degradation within 40 min and maintains high efficiency over repeated cycles, indicating good stability. Radical trapping experiments reveal that h^+^ and ·OH are the main reactive species, with ·O_2_^−^ also contributing, and that appropriate La doping promotes charge separation and suppresses recombination.

In summary, the combination of mesoporous confinement and optimal rare-earth doping offers a promising strategy for the design of robust photocatalytic systems aimed at the efficient degradation of organic pollutants in aqueous environments.

## Figures and Tables

**Table 1:** The mesoscopic structure parameters of the SBA-15, ZnO/SBA-15, and 5% La-ZnO/SBA-15.

Samples	S_BET_ (m^2^/g)	Pore Volume (cm^3^/g)	Pore Diameter (nm)
SBA-15	729.35	1.09	5.99
ZnO/SBA-15	534.61	0.90	6.74
5%La-ZnO/SBA-15	521.32	0.85	6.55

**Table 2:** Comparison with various ZnO-based composite photocatalysts.

Catalyst	Light Source	Dye Concertation	Photocatalyst Mass	Irradiation Time (min)	Efficiency (%)	Reference
Y doped V-ZnO NPs	Visible Light	RhB	3 g/L	180	87.5	[9]
ZnO-SiO_2_	UV Light	RhB (10 mg/L)	0.5 g/L	60	95	[37]
ZnO@SiO_2_	UV Light	RhB (20 mg/L)	15 g/L	180	82.5	[38]
Ru-induced ZnO/SBA-15	UV Light	MB (20 mg/L)	1 g/L	120	97.96	[39]
La-doped ZnO/SiO_2_	Sunlight	MG (15 mg/L)	0.3 g/L	120	92.1	[40]
ZnO/r-GO	Visible Light	RhB	0.5 g/L	150	100	[41]
SnO_2_/ZnO@GO	Visible Light	RhB (15 mg/L)	0.2 g/L	60	98.9	[42]
La-Doped ZnO/SBA-15	Visible Light	RhB (20 mg/L)	0.5 g/L	40	99.71	This work

## Data Availability

Data are contained within the article.

## References

[1] Nidheesh P.V., Couras C., Karim A.V., Nadais H. (2022). A review of integrated advanced oxidation processes and biological processes for organic pollutant removal. Chem. Eng. Commun..

[2] Titchou F.E., Zazou H., Afanga H., El Gaayda J., Ait Akbour R., Nidheesh P.V., Hamdani M. (2021). Removal of organic pollutants from wastewater by advanced oxidation processes and its combination with membrane processes. Chem. Eng. Process.—Process Intensif..

[3] Sharma D.K., Shukla S., Sharma K.K., Kumar V. (2022). A review on ZnO: Fundamental properties and applications. Mater. Today Proc..

[4] Zhou Z., Yang W., Peng H., Zhao S. (2024). Preparation and the Photoelectric Properties of ZnO-SiO_2_ Films with a Sol–Gel Method Combined with Spin-Coating. Sensors.

[5] Wang J., Chen R., Xiang L., Komarneni S. (2018). Synthesis, properties and applications of ZnO nanomaterials with oxygen vacancies: A review. Ceram. Int..

[6] Ibrahim M.W., Al-Obaidi M.A., Kosslick H., Schulz A. (2021). Photocatalytic degradation of pharmaceutical pollutants using zinc oxide supported by mesoporous silica. J. Sol-Gel Sci. Technol..

[7] Kankala R.K., Han Y.-H., Na J., Lee C.-H., Sun Z., Wang S.-B., Kimura T., Ok Y.S., Yamauchi Y., Chen A.-Z. (2020). Nanoarchitectured Structure and Surface Biofunctionality of Mesoporous Silica Nanoparticles. Adv. Mater..

[8] Zheng A.L.T., Abdullah C.A.C., Chung E.L.T., Andou Y. (2023). Recent progress in visible light-doped ZnO photocatalyst for pollution control. Int. J. Environ. Sci. Technol..

[9] Alam U., Khan A., Raza W., Khan A., Bahnemann D., Muneer M. (2017). Highly efficient Y and V co-doped ZnO photocatalyst with enhanced dye sensitized visible light photocatalytic activity. Catal. Today.

[10] Zhao D., Feng J., Huo Q., Melosh N., Fredrickson G.H., Chmelka B.F., Stucky G.D. (1998). Triblock Copolymer Syntheses of Mesoporous Silica with Periodic 50 to 300 Angstrom Pores. Science.

[11] Soltani S., Khanian N., Rashid U., Yaw Choong T.S. (2020). Fundamentals and recent progress relating to the fabrication, functionalization and characterization of mesostructured materials using diverse synthetic methodologies. RSC Adv..

[12] Wahab M.A., Beltramini J.N. (2015). Recent advances in hybrid periodic mesostructured organosilica materials: Opportunities from fundamental to biomedical applications. RSC Adv..

[13] Busuioc A.M., Meynen V., Beyers E., Mertens M., Cool P., Bilba N., Vansant E.F. (2006). Structural features and photocatalytic behaviour of titania deposited within the pores of SBA-15. Appl. Catal. A Gen..

[14] Abubakar Abdulkadir B., Mohd Zaki R.S.R., Abd Jalil A., Fang Su J., Setiabudi H.D. (2025). Synergistic effects of Fe_2_O_3_ supported on dendritic fibrous SBA-15 for superior photocatalytic degradation of methylene blue. Mater. Sci. Semicond. Process..

[15] Yuan S., Wang M., Liu J., Guo B. (2020). Recent advances of SBA-15-based composites as the heterogeneous catalysts in water decontamination: A mini-review. J. Environ. Manag..

[16] Lucovsky G., Phillips J.C. (2005). Defects and defect relaxation at internal interfaces between high-k transition metal and rare earth dielectrics and interfacial native oxides in metal oxide semiconductor (MOS) structures. Thin Solid Film..

[17] Hemalatha P., Karthick S.N., Hemalatha K.V., Yi M., Kim H.-J., Alagar M. (2016). La-doped ZnO nanoflower as photocatalyst for methylene blue dye degradation under UV irradiation. J. Mater. Sci. Mater. Electron..

[18] Vo V., Tran Thi T.P., Kim H.-Y., Kim S.J. (2014). Facile post-synthesis and photocatalytic activity of N-doped ZnO–SBA-15. J. Phys. Chem. Solids.

[19] Bariki R., Das K., Pradhan S.K., Prusti B., Mishra B.G. (2022). MOF-Derived Hollow Tubular In_2_O_3_/MIIIn_2_S_4_ (MII: Ca, Mn, and Zn) Heterostructures: Synergetic Charge-Transfer Mechanism and Excellent Photocatalytic Performance to Boost Activation of Small Atmospheric Molecules. ACS Appl. Energy Mater..

[20] Chae S., Yu J., Oh J.Y., Lee T.I. (2019). Hybrid poly (3-hexylthiophene) (P3HT) nanomesh/ZnO nanorod p-n junction visible photocatalyst for efficient indoor air purification. Appl. Surf. Sci..

[21] Lei Y., Yu Y., Lei X., Liang X., Cheng S., Ouyang G., Yang X. (2023). Assessing the Use of Probes and Quenchers for Understanding the Reactive Species in Advanced Oxidation Processes. Environ. Sci. Technol..

[22] Chen J., Feng Z., Ying P., Li M., Han B., Li C. (2004). The visible luminescent characteristics of ZnO supported on SiO_2_ powder. Phys. Chem. Chem. Phys..

[23] Lu Q., Wang Z., Li J., Wang P., Ye X. (2009). Structure and Photoluminescent Properties of ZnO Encapsulated in Mesoporous Silica SBA-15 Fabricated by Two-Solvent Strategy. Nanoscale Res. Lett..

[24] Wang Y.M., Wu Z.Y., Shi L.Y., Zhu J.H. (2005). Rapid Functionalization of Mesoporous Materials: Directly Dispersing Metal Oxides into As-Prepared SBA-15 Occluded with Template. Adv. Mater..

[25] Vepřek S., Iqbal Z., Sarott F.A. (1982). A thermodynamic criterion of the crystalline-to-amorphous transition in silicon. Philos. Mag. B.

[26] Mihai G.D., Meynen V., Mertens M., Bilba N., Cool P., Vansant E.F. (2010). ZnO nanoparticles supported on mesoporous MCM-41 and SBA-15: A comparative physicochemical and photocatalytic study. J. Mater. Sci..

[27] Sousa A., Sousa E.M.B. (2006). Influence of synthesis temperature on the structural characteristics of mesoporous silica. J. Non-Cryst. Solids.

[28] Ellerbrock R., Stein M., Schaller J. (2022). Comparing amorphous silica, short-range-ordered silicates and silicic acid species by FTIR. Sci. Rep..

[29] Khan S.A., Khan S.B., Khan L.U., Farooq A., Akhtar K., Asiri A.M., Sharma S.K. (2018). Fourier Transform Infrared Spectroscopy: Fundamentals and Application in Functional Groups and Nanomaterials Characterization. Handbook of Materials Characterization.

[30] Guo Y., Cheng M., Cui Y., Zhang R., Zhao Z., Wang X., Guo S. (2023). Effect of SBA-15-CEO on properties of potato starch film modified by low-temperature plasma. Food Biosci..

[31] Acosta-Silva Y.J., Nava R., Hernández-Morales V., Macías-Sánchez S.A., Gómez-Herrera M.L., Pawelec B. (2011). Methylene blue photodegradation over titania-decorated SBA-15. Appl. Catal. B Environ..

[32] Oréfice R.L., Vasconcelos W.L. (1997). Sol-Gel transition and structural evolution on multicomponent gels derived from the alumina-silica system. J. Sol-Gel Sci. Technol..

[33] Liang P., Yang W., Peng H., Zhao S. (2024). Efficient Degradation of Methylene Blue in Industrial Wastewater and High Cycling Stability of Nano ZnO. Molecules.

[34] Thommes M., Kaneko K., Neimark A.V., Olivier J.P., Rodriguez-Reinoso F., Rouquerol J., Sing K.S.W. (2015). Physisorption of gases, with special reference to the evaluation of surface area and pore size distribution (IUPAC Technical Report). Pure Appl. Chem..

[35] Abelniece Z., Kampars V., Piirsoo H.-M., Mändar H., Tamm A. (2022). The influence of Zn content in Cu/ZnO/SBA-15/kaolinite catalyst for methanol production by CO_2_ hydrogenation. Energy Rep..

[36] Saratovskii A.S., Bulyga D.V., Evstrop’ev S.K., Antropova T.V. (2022). Adsorption and Photocatalytic Activity of the Porous Glass–ZnO–Ag Composite and ZnO–Ag Nanopowder. Glass Phys. Chem..

[37] Hao Y.-Y., Zhang Y., Zhao L. (2017). Fabrication of ZnO–SiO_2_ and Its Photocatalytic Degradation of Rhodamine B. J. Synth. Cryst..

[38] Shen X., Shi Y., Shao H., Liu Y., Zhai Y. (2020). Synthesis and photocatalytic degradation ability evaluation for rhodamine B of ZnO@SiO_2_ composite with flower-like structure. Water Sci. Technol..

[39] Mihai S., Cursaru D.L., Şomoghi R., Nistor C.L. (2023). Synthesis of Ruthenium-Promoted ZnO/SBA-15 Composites for Enhanced Photocatalytic Degradation of Methylene Blue Dye. Polymers.

[40] Wang S., Chen Z., Zhao Y., Sun C., Li J. (2021). High photocatalytic activity over starfish-like La-doped ZnO/SiO_2_ photocatalyst for malachite green degradation under visible light. J. Rare Earths.

[41] Ding Y., Qin W., Zhu H., Dai Y., Hong X., Han S., Xie Y. (2025). Construction of ZnO/r-GO Composite Photocatalyst for Improved Photodegradation of Organic Pollutants. Molecules.

[42] Chen P., Li J., Wang J., Deng L. (2024). Synergistic Enhancement of Carrier Migration by SnO_2_/ZnO@GO Heterojunction for Rapid Degradation of RhB. Molecules.

[43] Zhao S., Yang L., Wang L., Yu B., Chen Y., Cui Y. (2010). Synthesis and Luminescence Properties of ZnO:Eu^3+^ Nanowire Arrays via Electrodeposited Method. Funct. Mater. Lett..

[44] Zhao S., Wang L., Yang L., Wang Z. (2010). Synthesis and luminescence properties of ZnO:Tb^3+^ nanotube arrays via electrodeposited method. Phys. B Condens. Matter.

[45] Zhao S., Ma H., Wang L., Yang L., Cui Y. (2010). Synthesis and Luminescence Properties of ZnO Nanoneedle Arrays via Electrodeposited Method. Surf. Rev. Lett..

[46] Moafi H.F., Zanjanch M.A., Shojaie A.F. (2014). Lanthanum and Zirconium Co-Doped ZnO Nanocomposites: Synthesis, Characterization and Study of Photocatalytic Activity. J. Nanosci. Nanotechnol..

[47] Zhou Y., Chen G., Yu Y., Zhao L., Yu Q., He Q. (2016). Effects of La-doping on charge separation behavior of ZnO:GaN for its enhanced photocatalytic performance. Catal. Sci. Technol..

[48] González-Crisostomo J.C., López-Juárez R., Petranovskii V. (2021). Photocatalytic Degradation of Rhodamine B Dye in Aqueous Suspension by ZnO and M-ZnO (M = La^3+^, Ce^3+^, Pr^3+^ and Nd^3+^) Nanoparticles in the Presence of UV/H_2_O_2_. Processes.

[49] Zhang X., Wang H., Shi Q., Zhang X., Jiang W., Lin X., Hu R., Liu T., Jiang X. (2025). Synergistic Optimization of Charge Carrier Separation and Transfer in ZnO through Crystal Facet Engineering and Piezoelectric Effect. J. Colloid Interface Sci..

[50] Sun N., Si X., He L., Zhang J., Sun Y. (2024). Strategies for Enhancing the Photocatalytic Activity of Semiconductors. Int. J. Hydrogen Energy.

